# Planar Quadrature RF Transceiver Design Using Common-Mode Differential-Mode (CMDM) Transmission Line Method for 7T MR Imaging

**DOI:** 10.1371/journal.pone.0080428

**Published:** 2013-11-12

**Authors:** Ye Li, Baiying Yu, Yong Pang, Daniel B. Vigneron, Xiaoliang Zhang

**Affiliations:** 1 Department of Radiology and Biomedical Imaging, University of California San Francisco, San Francisco, California, United States of America; 2 Paul C. Lauterbur Research Center for Biomedical Imaging, Shenzhen Key Laboratory for MRI, Institute of Biomedical and Health Engineering, Shenzhen Institutes of Advanced Technology, Chinese Academy of Sciences, Shenzhen, Guangdong, China; 3 Magwale, Palo Alto, California, United States of America; 4 UC Berkeley/UCSF Joint Graduate Group in Bioengineering, Berkeley & San Francisco, California, United States of America; 5 California Institute for Quantitative Biosciences (QB3), San Francisco, California, United States of America; University of Minnesota, United States of America

## Abstract

The use of quadrature RF magnetic fields has been demonstrated to be an efficient method to reduce transmit power and to increase the signal-to-noise (SNR) in magnetic resonance (MR) imaging. The goal of this project was to develop a new method using the common-mode and differential-mode (CMDM) technique for compact, planar, distributed-element quadrature transmit/receive resonators for MR signal excitation and detection and to investigate its performance for MR imaging, particularly, at ultrahigh magnetic fields. A prototype resonator based on CMDM method implemented by using microstrip transmission line was designed and fabricated for 7T imaging. Both the common mode (CM) and the differential mode (DM) of the resonator were tuned and matched at 298MHz independently. Numerical electromagnetic simulation was performed to verify the orthogonal B_1_ field direction of the two modes of the CMDM resonator. Both workbench tests and MR imaging experiments were carried out to evaluate the performance. The intrinsic decoupling between the two modes of the CMDM resonator was demonstrated by the bench test, showing a better than -36 dB transmission coefficient between the two modes at resonance frequency. The MR images acquired by using each mode and the images combined in quadrature showed that the CM and DM of the proposed resonator provided similar B_1_ coverage and achieved SNR improvement in the entire region of interest. The simulation and experimental results demonstrate that the proposed CMDM method with distributed-element transmission line technique is a feasible and efficient technique for planar quadrature RF coil design at ultrahigh fields, providing intrinsic decoupling between two quadrature channels and high frequency capability. Due to its simple and compact geometry and easy implementation of decoupling methods, the CMDM quadrature resonator can possibly be a good candidate for design blocks in multichannel RF coil arrays.

## Introduction

Signal-to-noise ratio (SNR) and transmit efficiency are two fundamental considerations for radio frequency (RF) coil design in magnetic resonance (MR) imaging. Circularly-polarized or quadrature transmit/receive coils have been demonstrated to be an efficient method to increase the SNR and to reduce the signal excitation power [1-5]. In volume RF coil, such as birdcage coils, quadrature RF fields (B_1_ fields) can be realized by driving two orthogonal ports with equal amplitude and a 90 degree phase difference [6-12]. There are several approaches to construct quadrature surface coils. One common design consists of two identical surface coils placed orthogonally [1,13,14]. In order to construct planar quadrature coils for MR imaging, two planar surface coils with different structures capable of generating orthogonal B_1_ field, are utilized to obtain quadrature B_1_ fields [15-20].

Electromagnetic coupling between two quadrature modes or channels is a critical issue in quadrature RF coil designs and also a major cause of SNR degradation in MRI acquisitions. In ultrahigh field MRI, this coupling issue becomes amplified because of increased electromagnetic interaction at high frequency. In addition, the conventional RF coils using lumped elements for human imaging show limitation in achieving the high resonant frequency at ultrahigh fields. Recent studies of double tuned coil designs using the common-mode differential mode (CMDM) method indicate that the common-mode (CM) and differential-mode (DM) provide two orthogonal and intrinsically decoupled B_1_ fields by the two current modes in one single CMDM resonator [21-23]. This characteristic of CMDM resonators would be advantageous to generate high performance quadrature fields when its two modes are tuned to the same frequency [24] if the decoupling property still holds.

In this work, we design and investigate the quadrature surface resonator based on CMDM technique using transmission lines for ultrahigh field MR imaging. Numerical simulation based on Finite-Difference Time-Domain (FDTD) algorithm was employed to investigate the orthogonal B_1_ field direction of the two modes of the CMDM resonator. A prototype CMDM coil was designed and fabricated using the microstrip transmission line [25], which is proven to be advantageous to ultrahigh field RF coil designs due to its high quality factor, high frequency operation capability and high efficiency [7,9,25-36]. Workbench tests were carried out to measure the resonance frequency, impedance matching and intrinsic decoupling performance of the two modes. MR imaging of a water phantom was performed to investigate coil performance. Due to its unique structure, CMDM provides a practical and convenient method for building compact planar quadrature coils, and also can possibly be a good candidate for design blocks in quadrature phased arrays.

## Materials and Methods

### CMDM Coil Design and Fabrication

**Figure 1:**
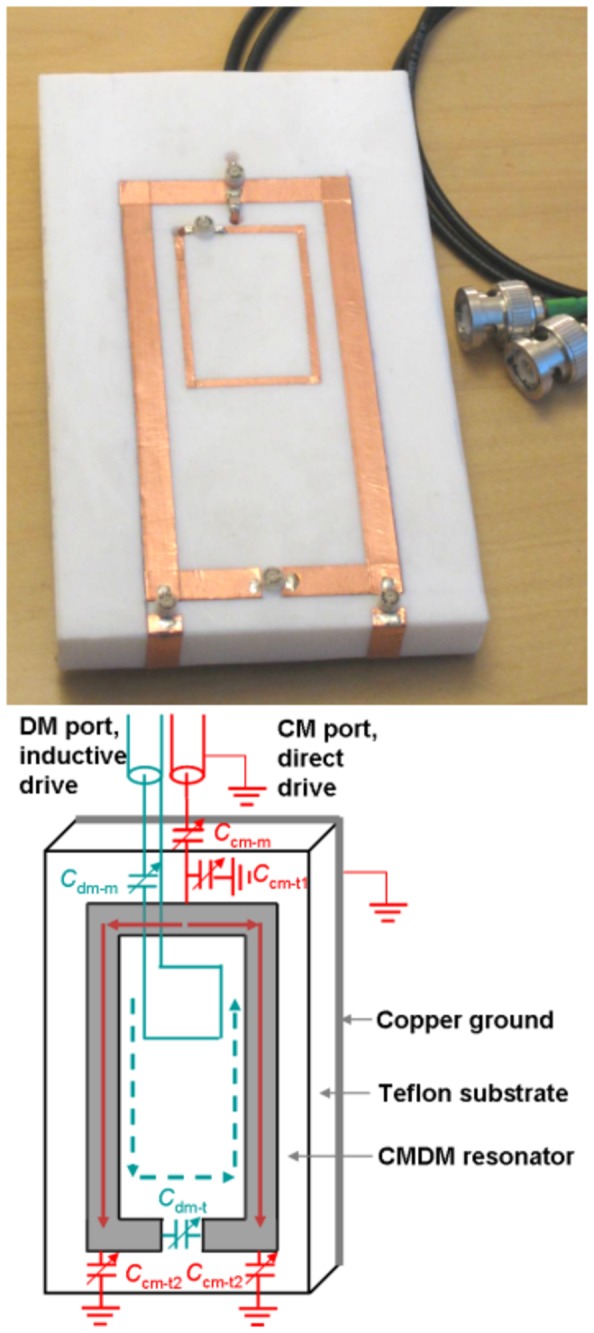
Prototype coil and configuration of the compact planar CMDM quadrature coil. Dimensions of the resonator were 9.4cm x 4.3cm. The resonator was built on Teflon substrate with copper foil on the opposite side serving as ground. The red solid line indicates the common mode current, which was directly driven by coaxial cable. The blue dashed line indicates the differential mode current, which was inductively driven by a small loop in the center of the resonator.

As shown in Figure 1, the dedicated planar CMDM quadrature coil with 4.3cm width and 9.4cm length was fabricated with 6.35mm width copper strips on 1.27cm thickness Teflon substrate. The opposite side of the Teflon substrate was covered by copper foil serving as a ground plane. This layout formed a symmetrically 180-degree bent microstrip resonator. The CM was the second harmonic mode of the microstrip resonator and was driven directly by a coaxial cable at the center of the microstrip. In this mode, the currents on the two parallel strip conductors of the microstrip resonator are in the same direction. The CM was tuned by a 91 pF terminated capacitor (American Technical Ceramics Corp., Huntington, NY) on the driven port, which is indicated as *C*_cm-t1_, and trimmer capacitors C_cm-t2_ = 0.5 - 2.5 pF as shown in Figure 1. The matching trimmer capacitor of CM was C_cm-m_ = 2.5 - 10 pF. Both the shield of the red coaxial cable and the copper ground plane are grounded. The DM was the primary resonance mode or first harmonic of the microstrip resonator and was driven inductively by a 2.6cm × 3.9cm square loop with a trimmer matching capacitor C_dm-m_ = 2.5 - 10 pF. The loop was fabricated by 0.16cm width copper tape. In this mode, the currents on the two parallel strip conductors of the microstrip resonator are in opposite direction. The DM was tuned by trimmer capacitors C_dm-t_ = 0.5 - 2.5 pF as shown in Figure 1. All the trimmer capacitors were from Johanson Manufacturing Corp. (Booton, NJ). 

The two modes were tuned to 298 MHz corresponding to the proton Larmor frequency at 7T and matched to 50 Ohm. Workbench tests on the resonance modes and their isolation were carried out on the network analyzer E5070B (Agilent, Santa Clara, CA). 

### FDTD Simulations

 FDTD simulation using XFDTD 6.5 (Remcom, State College, PA) was utilized to investigate the B_1_ field distribution of the two modes of the CMDM resonator in the unloaded and loaded (with water phantom and with human head model) cases. The relative permittivity of the Teflon substrate and water phantom was set to 2.1 and 78 respectively. The dimensions of water phantom were 120mm × 75mm × 75mm. The conductor of the resonator was modeled as thin copper foils with conductivity of 5.7×107 S/m. The simulations were performed for three different driven conditions: CM, DM (unloaded case) and the two modes simultaneously driven by equal amplitude with a 90 degree phase difference (all three unloaded and loaded cases). A sine wave at 298 MHz was used as the current source to excite the resonator. The convergence threshold was set to -60 dB.

### MR Imaging Experiments

MR imaging experiments were performed on a General Electric (GE) whole body 7T scanner. MR images were acquired from a water phantom using the proposed CMDM quadrature resonator. The two modes of the CMDM quadrature resonator were driven in equal amplitude with a 90 degree phase difference for the excitation of the MRI experiments. The signals from two modes were acquired simultaneously. The water phantom images were acquired by using gradient echo (GRE) image sequence with the parameters: flip angle = 30°; TE = 5 ms; TR = 800 ms; slice thickness = 5 mm; FOV = 14 cm × 14 cm; 256×256 image matrix; number of excitation (NEX) = 1. The SNR of CM, DM and quadrature mode are calculated and compared to evaluate the coil performance. Since the excitation of the three modes is the same, the SNR comparison is not affected by the excitation profiles. The SNR is given by [37]

*SNR = 0.655 Signal / SD_air_* (1)

where Signal denotes the image intensity of each pixel and SD_air_ is the standard deviation of two small regions placed on the background air pixels. The background regions were chosen to avoid ghosting and reconstruction artifacts. In order to quantify the effective quadrature region, the images of quadrature case, anti-quadrature case were both evaluated; and the SNR ratio of the quadrature and anti-quadrature cases was calculated.

## Results

### FDTD Simulations

**Figure 2:**
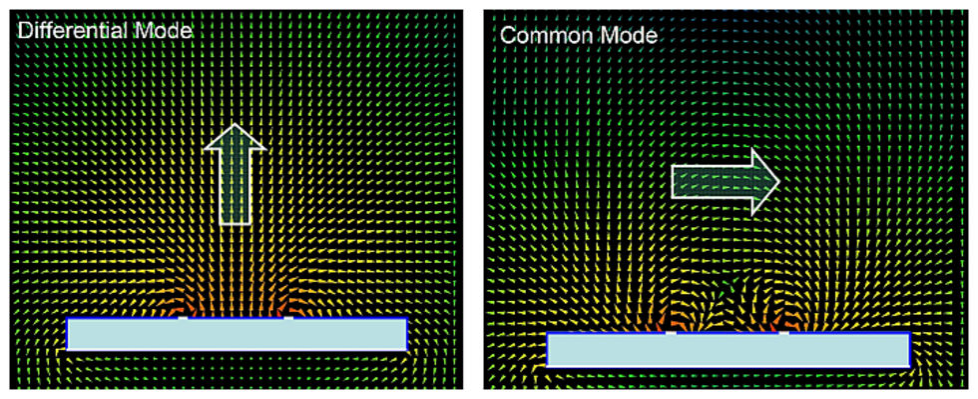
FDTD simulation results of the B_1_ field distribution of the differential mode and common mode. The two modes were driven respectively by the current source with equal amplitude and the same phase. The white lines indicate the strip conductors and the ground plane. The blue boxes indicate Teflon substrates. The white arrows indicate the B_1_ field direction, demonstrating the orthogonal B_1_ fields of the two modes. Differential mode was inductively driven by using a small loop in the center of the resonator.

**Figure 3:**
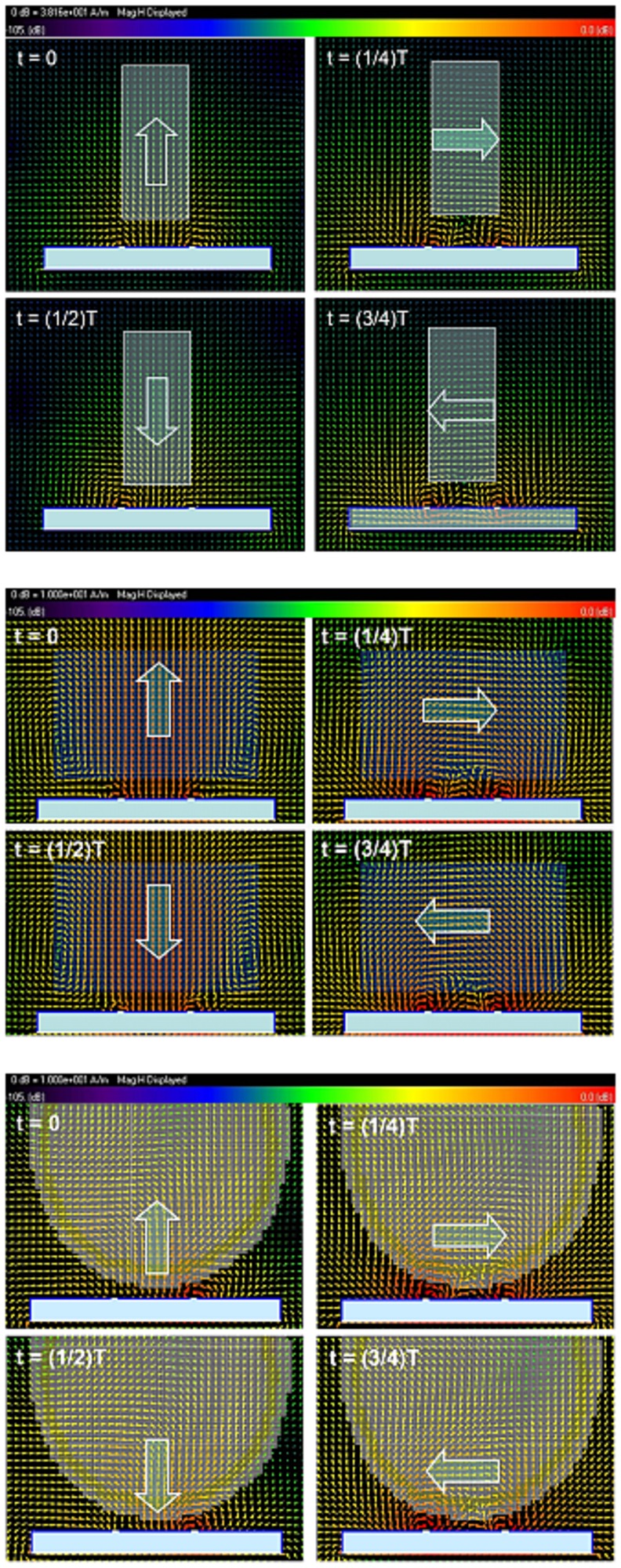
FDTD simulation results of the CMDM quadrature coil in: (a) unloaded case, (b) loaded case with water phantom and (c) loaded case with human head model. The two modes were driven simultaneously by current sources with equal amplitude and a 90 degree phase difference. The B_1_ field distribution at 4 moments in one period T demonstrates the orthogonal B_1_ fields of the CMDM quadrature resonator.

FDTD simulation results of the B_1_ field distribution of the differential mode and common mode of the unloaded CMDM quadrature resonator are shown in Figure 2. In this case, the two modes were driven respectively by current source with equal amplitude and the same phase. The white solid lines indicate the resonator and the copper ground and the blue boxes indicate Teflon substrates. The white arrows show the B_1_ field directions. The simulation results verify the orthogonal B_1_ field of the two modes. Figure 3 shows the B_1_ field distribution of the CMDM quadrature coil in following three cases -(a) unloaded, (b) loaded with water phantom and (c) loaded with human head model. The two modes were driven simultaneously by current sources with equal amplitude and 90 degree phase difference. The B_1_ field distribution at 4 moments in one period T (i.e., t = 0, 0.25T, 0.5T and 0.75T) demonstrate the quadrature behavior of the B_1_ fields of the CMDM resonator. Simulation results showed that S21 of the two modes was better than -70 dB in all three loaded or unloaded cases, indicating the intrinsic decoupling of the two modes which is essential for quadrature coils. 

### Workbench Study

**Figure 4:**
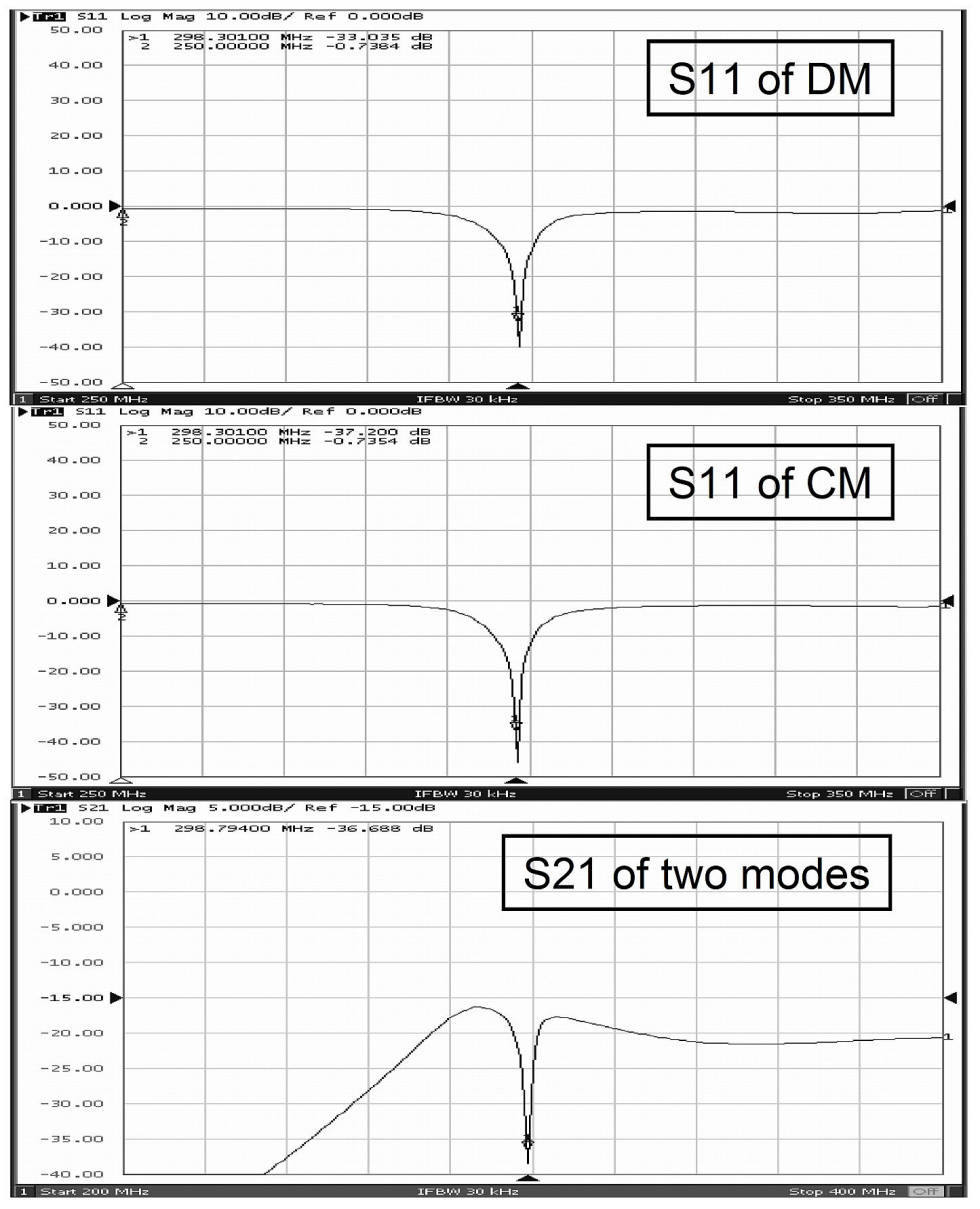
Scattering coefficients S11 and S21 of the planar CMDM quadrature resonator. (a) S11 = -33 dB of the differential mode; (b) S11 = -37 dB of the common mode; (c) S21 = -36.7 dB of the two modes. A better than -36 dB isolation between the two modes was achieved, which verified the intrinsic decoupling character of the two modes in the CMDM resonator.

The reflection coefficient S11 and the transmission coefficient S21 of the two modes of the proposed CMDM quadrature resonator are measured by using the network analyzer. As shown in Figure 4, the S11 of differential mode is achieved -33 dB and that of common mode reaches -37 dB. The results indicated an excellent impedance matching of the CMDM coil. The measured S21 of two modes is better than -36 dB, which demonstrates the deep decoupling of the two modes. The S21 difference between simulation and workbench results might be caused by unsymmetrical coil fabrication. Unloaded and loaded Q factors of CM are 341 and 147 respectively while those of DM are 323 and 155, respectively.

### MR Imaging Experiment

**Figure 5:**
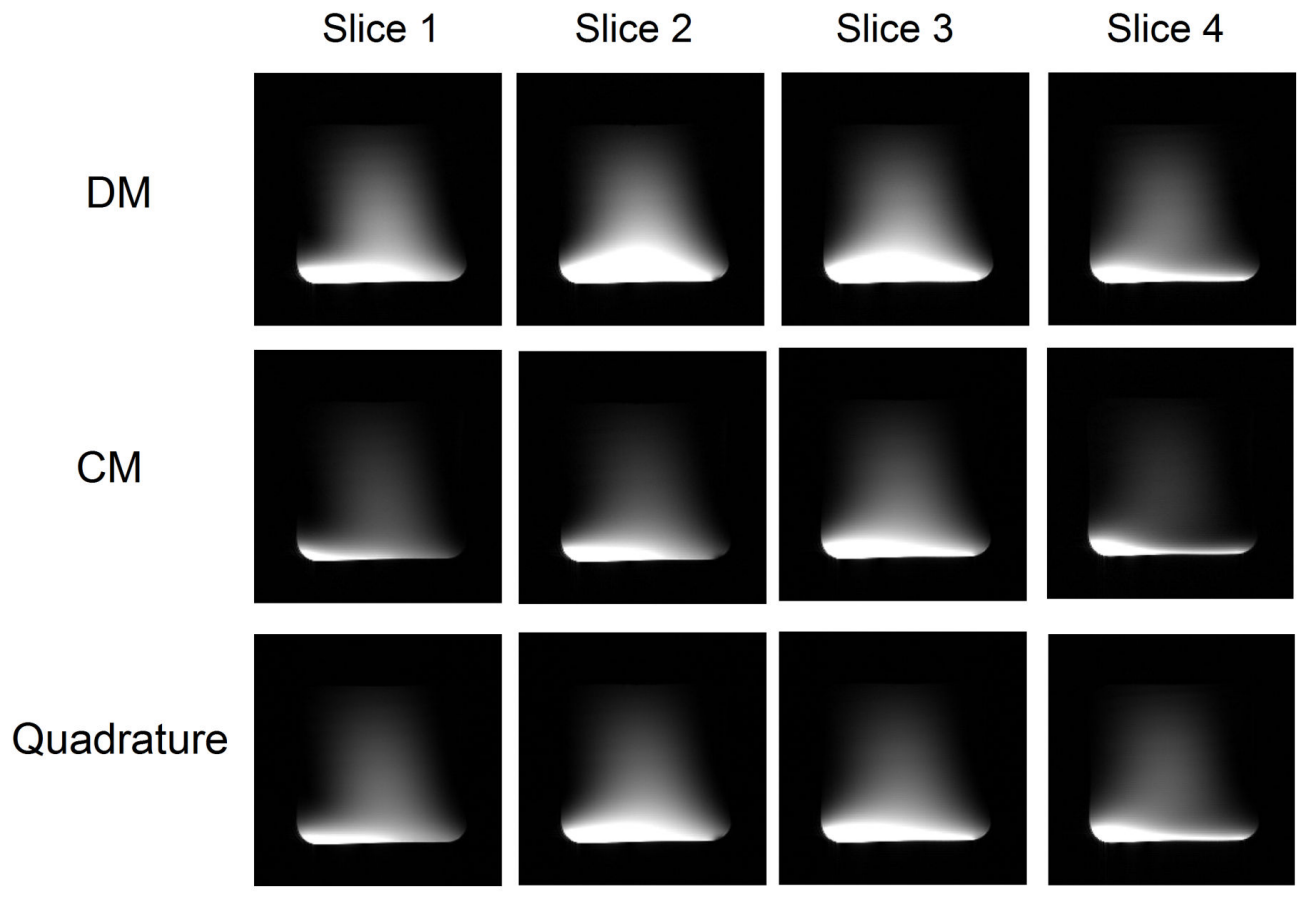
MR imaging experiments. MR images in the sagittal direction acquired from a water phantom by using the CMDM quadrature resonator at 7T: differential mode (top row), common mode (middle row) and quadrature mode (bottom row). Imaging parameters are: flip angle = 30°; TE = 5 ms; TR = 800 ms; slice thickness = 5 mm; FOV = 14 cm × 14 cm; 256×256 image matrix; number of excitation (NEX) = 1. The two modes of the resonator show similar imaging coverage, which helps to gain improved quadrature performance.

**Figure 6:**
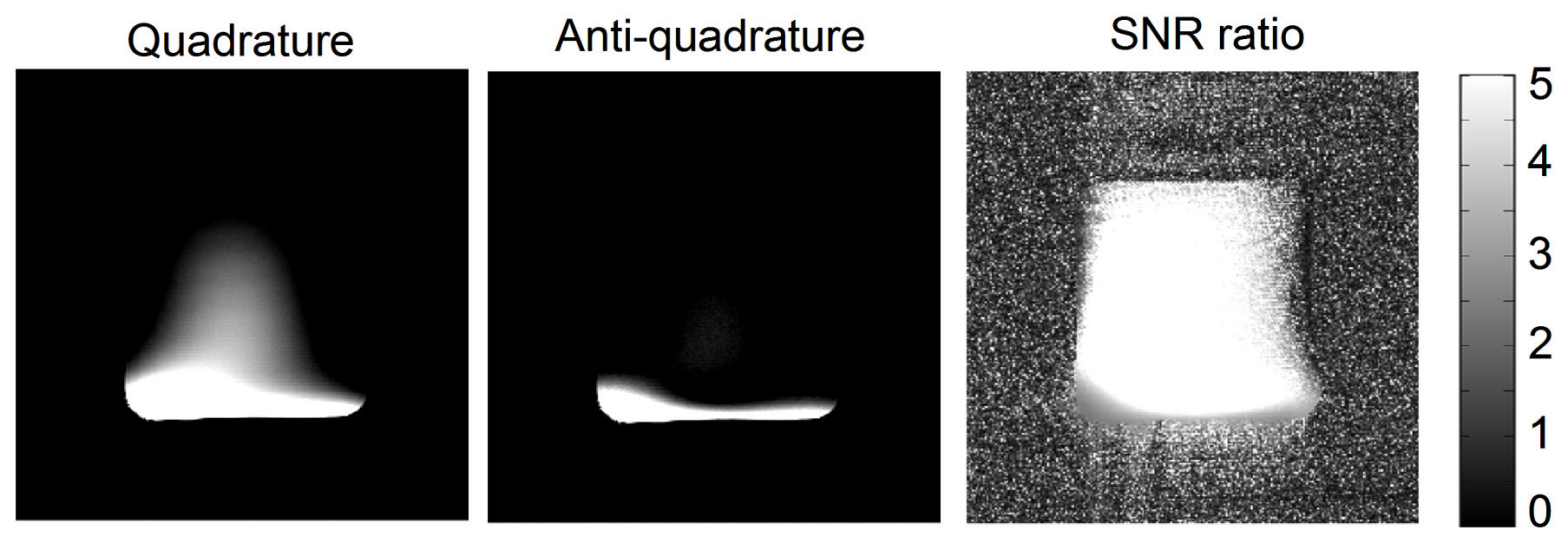
The quadrature behavior of the CMDM method. The images of quadrature case, anti-quadrature case, and the map of the ratio of quadrature image to anti-quadrature image (right insert) indicating the effective quadrature region. The white color in the ratio map indicates the numbers equal or greater than 5.

Figure 5 shows the MR images of the water phantom acquired using the common mode, differential mode and the quadrature mode of the proposed quadrature CMDM resonator at 7T. The images show that the CM and DM of the quadrature coil possess similar B_1_ distribution and coverage, which helps provide quadrature fields in the nearly entire coil sensitivity region. The average SNR of the quadrature mode increased approximately 24% in the image region compared with the higher SNR between CM and DM. Figure 6 shows the images of quadrature case, anti-quadrature case, and the map of the ratio of the quadrature image to the anti-quadrature image. The signal in most of the imaging region has been canceled in the anti-quadrature case except in the area close to the coil conductors. The results demonstrate reasonable quadrature performance and the effective quadrature region of this CMDM design.

## Discussion

The results of bench tests and MR imaging experiments demonstrate that the proposed CMDM method is a feasible and efficient technique for quadrature surface coil designs for ultra-high field MR imaging. The intrinsic decoupling between the common mode fields and differential mode fields, which is due to the symmetric geometries of the CMDM resonator and the orthogonal magnetic field distribution of the two modes, provides an important feature for quadrature RF coil design. In the CMDM design, SNR improvement resulting from the quadrature behavior can be realized in nearly the entire coil sensitivity region, except for the area adjacent to the coil conductors. The SNR gain can be further improved by optimizing the coil structure, such as the substrate thickness and coil dimensions. For instance, in the specific design shown in this work, reducing the coil width can increase the B_1_ strength of the common mode in the imaging area. This may improve the equality of the field strength of the common mode and differential mode, yielding “true” circular polarization (rather than an elliptical polarization), and consequently improve imaging SNR and excitation efficiency.

The proposed CMDM coil used in this study was implemented by using a microstrip transmission line resonator, which consists of both first and second harmonics. The benefits of using first harmonic and/or second harmonic microstrip transmission line (MTL) in RF coil design have been demonstrated previously [25,38-42]. In this design, the use of termination capacitors can reduce the phase variation to generate homogenous radiofrequency fields at ultrahigh fields. In the proposed CMDM coil, the tuning capacitor of the DM was employed to increase the tuning range. However it does not have to use the capacitor in the CMDM design. In fact, the CMDM coil can be built on a single U-shape MTL without the capacitor Cdm-t where its first harmonic provides differential-mode and the second harmonic gives common-mode. In this case, the frequency of DM mode can be tuned by capacitors Ccm-t2 and the frequency of CM mode can be tuned by the capacitor on the driven port. In addition, distributing capacitors over the current path can certainly be employed for lessening any potential phase variation of the current in the resonator.

In the proposed CMDM quadrature coil design, the differential mode was driven by using inductive coupling with a matching loop. The inductive coupling to RF coils has been widely used and analyzed [43-45]. As suggested in reference [45], the field modification caused by the matching loop is only the small remainder terms of order 2 if the two fields are parallel. Interference of the matching loop could be minimized by keeping the inductance of the coupling loop as small as possible [44].

In current phased array coil designs, the array elements made for phased array coils are usually linear surface coils. Due to their bulky structure and difficulties in achieving sufficient electromagnetic decoupling among the array elements, conventional quadrature surface coils are not readily to be used for phased array coils with quadrature capability for better SNR and reduced RF excitation power. The proposed CMDM quadrature surface coils have a regular and simple rectangular geometry and it is easy to implement the decoupling methods, particularly the ICE decoupling technique [28]. Therefore the CMDM quadrature coils might be a good choice as building blocks for quadrature phased array coils. 
